# A comparative study of cone beam computed tomography and conventional radiography in diagnosing the extent of root resorptions

**DOI:** 10.1186/s40510-017-0191-z

**Published:** 2017-11-20

**Authors:** Elham Alamadi, Hisham Alhazmi, Ken Hansen, Ted Lundgren, Julia Naoumova

**Affiliations:** 1 Specialist Clinic of Orthodontics, University Clinics of Odontology, Public Dental Service Västra Götaland Region, Gothenburg, Sweden; 2 Department of Pediatric Dentistry, Institute of Odontology at Sahlgrenska Academy, University of Gothenburg, Gothenburg, Sweden

## Abstract

**Background:**

Root resorptions are assessed and diagnosed using different radiographical techniques. A comparison of the ability to assess resorptions on two-dimensional (2D) and three-dimensional (3D) radiographs is, hitherto, lacking. The aims of this study were to evaluate the accuracy of 2D (periapical radiographs, PA and panoramic radiograph, PAN) and 3D (cone beam computed tomography, CBCT) radiographic techniques in measuring slanted root resorptions compared to the true resorptions, a histological gold standard, in addition to a comparison of all the radiographic techniques to each other.

**Methods:**

Radiographs (CBCT, PA, and PAN), in addition to histological sections, of extracted deciduous canines from thirty-four patients were analyzed. Linear measurements of the most and least resorbed side of the root, i.e., “slanted” resorptions, were measured using an analyzing software (Facad ®). For classification of slanted root resorptions, a modified Malmgren index was used.

**Results:**

PAN underestimated the root length on both the least and most resorbed side. Small resorptions, i.e., low modified Malmgren scores, were more difficult to record and were only assessed accurately using CBCT. The root resorption scores were underestimated using PA and PAN. In assessment of linear measures, PAN differed significantly from both CBCT and PA.

**Conclusions:**

CBCT is the most accurate technique when measuring and scoring slanted root resorptions.

## Background

Accurate diagnosis and assessment of the extent of root resorption is important in orthodontic treatment and treatment planning. The diagnosis of root resorptions mainly depends on radiographic examinations. Conventional two-dimensional (2D) radiographical techniques are the most commonly used methods for diagnosis of root resorption. These methods, however, have limitations such as superimposition of structures onto the two-dimensional plane, distortion projection errors, and blurred images [1–5]. Furthermore, the conventional radiographs are inadequate in grading resorption lesions and in detecting resorption dimensions [2, 6, 7].

During the 1990s, cone beam computed tomography (CBCT) was introduced and is now widely used. Studies have shown that CBCT imaging is a more reliable tool for detection of small resorptions compared to conventional panoramic (PAN) radiograph [8, 9].

When periapical (PA) radiographs were compared to CBCT images, root lengths were underestimated by an average of 2.6 mm compared to 0.3 mm underestimation in CBCT images. In a comparison of the accuracy of CBCT and PA radiographs in detecting small periapical resorptions, CBCT showed poor accuracy in detecting simulated resorptions smaller than 0.8 mm in diameter and excellent when the lesions were larger than 1.4 mm in diameter. On the other hand, PA radiographs demonstrated poor accuracy for all simulated resorption sizes. A higher accuracy was reported with PA radiographs when lesions were larger (2 to 5 mm) in diameter [10–13]. Linear measurement is usually used for measuring root length and the resorption level. Several studies have investigated the accuracy of linear measurements in CBCT. Findings indicate that these measurements are highly accurate [14–17].

Malmgren root resorption scoring system with index scores from 0 to 4 is commonly used to classify the severity of horizontal root resorption. In order to assess slanted root resorptions, a modified Malmgren index is needed [18]. A thorough comparison of various radiographic techniques and their ability to detect horizontal and slanted resorptions is, hitherto, lacking. To our knowledge, no study has compared slanted root resorptions on PA, PAN, or CBCT in relation to a true histological gold standard. The aims of this study were to evaluate the accuracy of three radiographic techniques: CBCT, PA, and PAN in measuring slanted root resorptions compared to a true histological gold standard, in addition to determining which technique is most precise in detecting slanted root resorption by comparing PA, PAN, and CBCT to each other.

## Methods

**Fig. 1:**
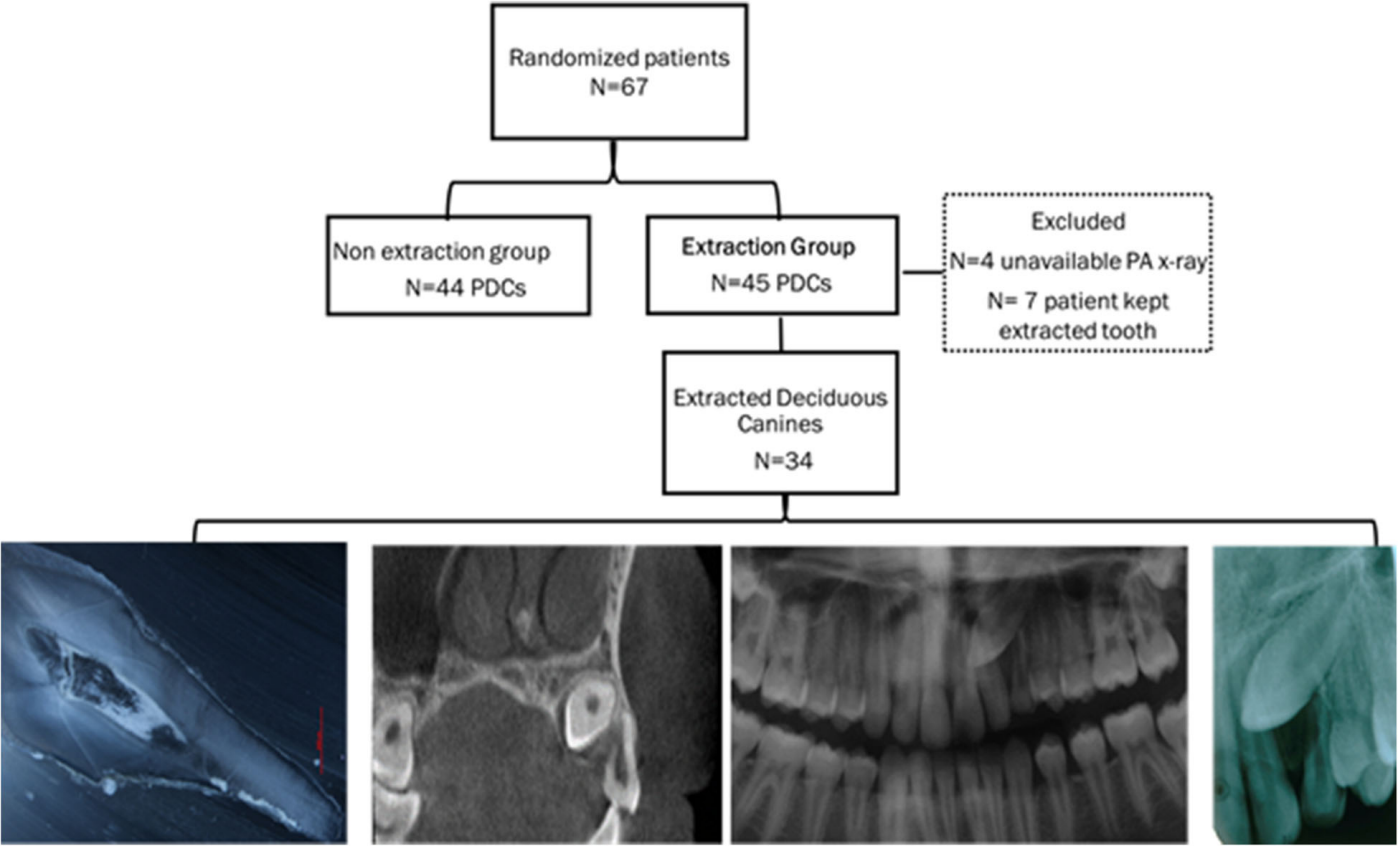
Flow chart describing the study material. *N* = amount of patients, PDCs = palatally displaced canines, PA = periapical radiographs

The patient in this study is from a previous prospective randomized trial on interceptive treatment of palatally displaced canines (PDCs) by extracting the deciduous canine. Details about the patients, their recruitment, inclusion, and exclusion criteria are described in this study [19]. Sixty-seven patients (40 girls, mean age ± SD 11.3 ± 1.1, 27 boys mean age ± SD 11.4 ± 0.9) with eighty-nine PDCs were randomized by block randomization method with allocation concealment in consecutive numbers of sealed envelopes. Patients were randomized to have either extraction of a deciduous canine or non-extraction. The extraction group was included in the present study, which consisted of forty-four children: 20 boys (11.5 ± 1.2) and 24 girls (11.2 ± 1.2), with forty-five PDCs. Eleven teeth were excluded due to either missing PA radiographs and due to patient’s wish to keep the extracted deciduous canine (Fig. 1).

The study was approved by the research ethics committee of Sahlgrenska Academy at the University of Gothenburg, Sweden, and by the radiation protection committee. Parents and patients have received verbal and written information, in addition to informed consent provided by the patient or the parent in accordance with the Declaration of Helsinki.

### Radiographic techniques

All patients had a set of radiographic images before extraction of deciduous canines. These radiographs included, at least, two PA, one PAN, and one CBCT. The PAN and CBCT were taken on the same day as the extraction of the deciduous canine, while the PA radiographs were taken not more than 3 months before the extraction.

PA radiographs were taken by the general practitioners in the public dental clinics, in bisecting angle and parallel techniques. At least two radiographs on each tooth in a different angle to detect the position of the permanent canine were taken. All PA radiographs were scanned in high resolution (2000dcm). PAN were taken at the Department of Oral and Maxillofacial Radiology at the Institute of Odontology at Sahlgrenska Academy, with 1.7-degree magnification (Scanora, Soredex, Finland). CBCT was performed at the Department of Oral and Maxillofacial Radiology at the Institute of Odontology at Sahlgrenska Academy, with 3D Accuitomo FPD (J. Morita, Kyoto, Japan) with a 360-degree rotation. The volume used was 60 × 60 mm. Primary data reconstructions were made by acquisition software (i-Dixel-3DX, 3D Version 1.691, J Morita Mfg Corp) at the Accuitomo workstation, providing axial, frontal, and sagittal views. Secondary reconstruction was then made using the i-Dixel software. A sagittal radiographical view of the deciduous canine was performed with a Sectra Imtec IDSS multiplanar reconstruction program with a reference line placed at the middle of the pulp chamber and another intersecting line with a 45-degree angle. This was made in order to obtain 3D CBCT images comparable to 2D periapical images.

### Histological measurements

**Fig. 2:**
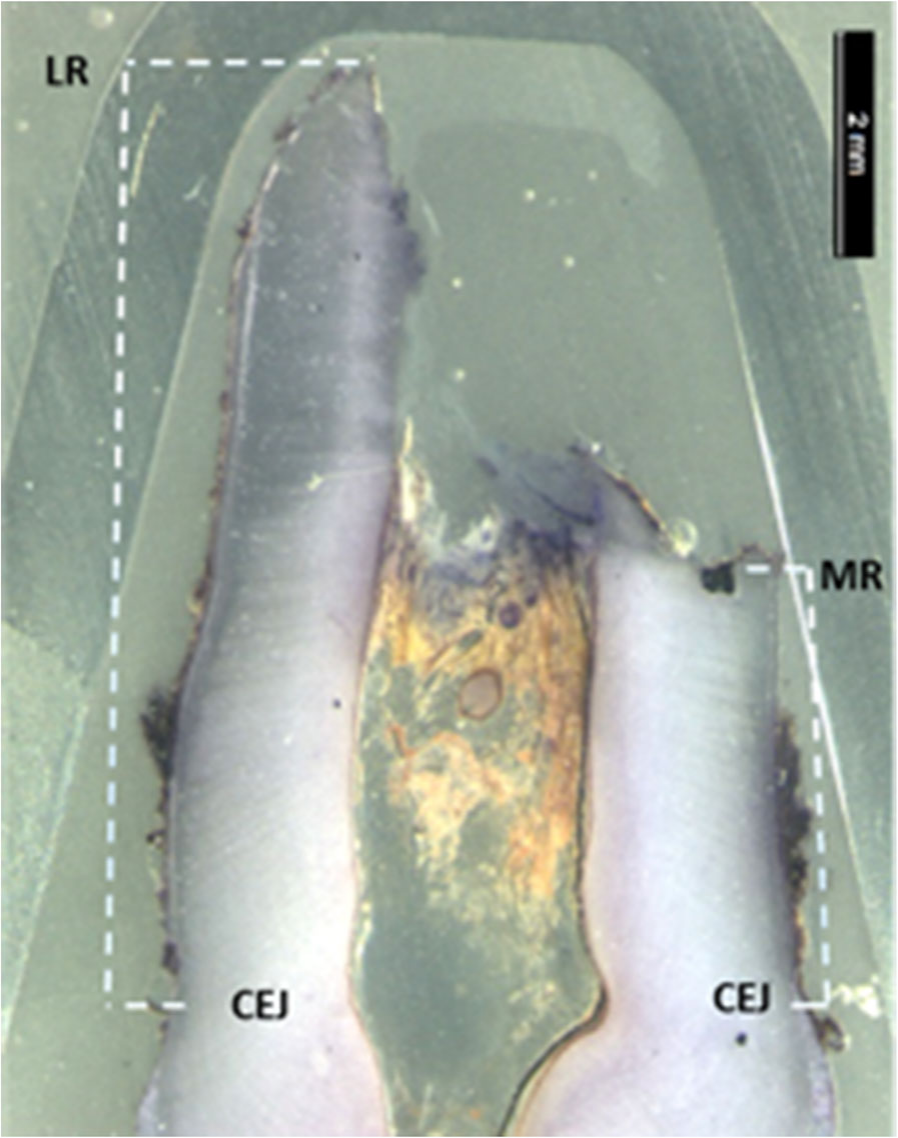
Microscopic image of an extracted deciduous canine showing slanted root resorption and how the root length measurements from cemento-enamel junction (CEJ) to the most resorbed (MR) side and from CEJ to the less resorbed (LR) root side were measured

Extracted deciduous teeth were stored in 5% buffered formaldehyde. Before embedding, the teeth were washed several times in 70% ethanol, with a final wash in absolute ethanol. Then, the teeth were embedded in epoxy-resin (Epofix®; Electron Microscopy Sciences, Fort Washington, PA), and each tooth was cut sagittally, in a bucco-lingual direction into halves in a Leitz low-speed saw microtome. From the central part of the teeth, three sections were cut with a thickness of 110 μm. All sections were photographed with fiber-optic surface illumination in a Nikon SMZ800 microscope equipped with a Nikon Digital Sight DS-F1 camera. The slanted root resorption evident on the most central cut of the tooth was used as gold standard (Fig. 2).

### Root resorption measurements

**Fig. 3:**
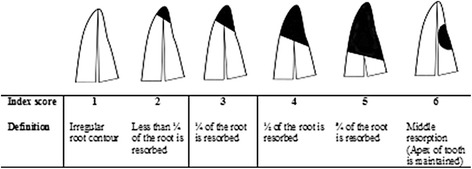
Modified Malmgren classification of slanted root resorption with definition

Radiographical (PA, PAN, and CBCT) and histological images of 34 patients, in total 136 images, were coded with a numeric sign, and the order of the images was randomized in www.random.org. The radiographical and histological images were imported into an analyzing software (Facad version 3.0, Ilexis AB, Linköping, Sweden) and were calibrated regarding magnification. Assessment of the most resorbed and the least resorbed root side was made on each image by two calibrated examiners (EA and HAL). Two linear measurements were done on each image. The most resorbed (MR) side of the root was defined as the distance from cemento-enamel junction to the most resorbed point on the root. The less resorbed (LR) side of the root was defined as the distance from the cemento-enamel junction to the least resorbed point on the root surface. This was considered a “slanted” resorption (Fig. 2). The original Malmgren index used for classification of root resorptions was completed with additional scores [18]. For classification of slanted root resorption, a modified Malmgren index, now comprising of six scores, was used (Fig. 3). When the apex of the tooth was intact, but a resorption was evident on the root side, the resorption was defined as “middle” resorption. Middle resorptions were measured from the most cervical resorption point to the most apical resorption point in a linear line (Fig. 3, score 6).

A first set of measurements was blinded; thus, root resorption measurements were performed by each operator on each radiographic image without comparing the image to the gold standard. In a second not blinded measurement set, the root resorption level in each radiographic image was compared to gold standard. The data from the blinded and not blinded measurement sets was analyzed by each operator in a 2 months’ interval period.

### Statistical analysis

Two root resorption measurements were performed for each tooth; one measured by examiner EA and the other by examiner HAL. The mean of the two measurements was used for the statistical analysis. Paired *t* tests were used to compare the blinded and the not blinded data sets. *p* values < 0.05 was considered statistically significant. The inter-examiner reliability was assessed with Cohen’s kappa with less than 5% deviation from the gold standard. The Malmgren classification of the gold standard sections was compared to the radiographic sections using Mann-Whitney test. All statistical tests were performed using the statistical package in Excel.

## Results

### Comparison of root length to gold standard

**Fig. 4:**
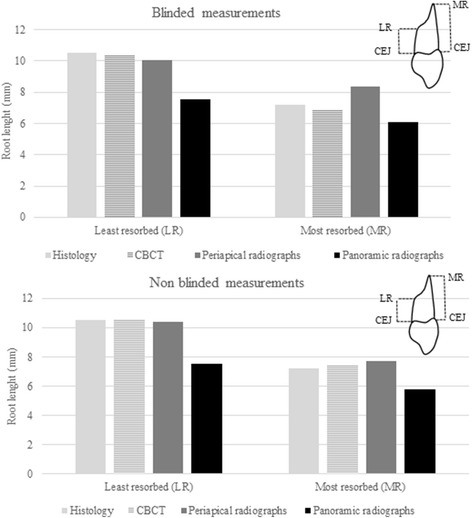
Blinded and not blinded measurements of the least resorbed (LR) and most resorbed (MR) side of the root in millimeters measured on histology (gold standard), cone beam computed tomography (CBCT), periapical radiographs, and panoramic radiograph (PAN). PAN differed significantly (*p* < 0.05) in both blinded and not blinded measurements compared to histology

For both blinded and not blinded measurements, PAN underestimated the root length on the least and most resorbed side. However, measurements with CBCT and PA were similar to gold standard (Fig. 4).

### Comparison of modified Malmgren classification to gold standard

**Fig. 5:**
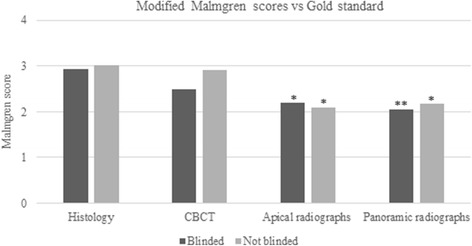
Modified Malmgren scores for CBCT, periapical radiographs (PA), and panoramic radiographs (PAN) versus gold standard (histology). PA and PAN differed significantly in both blinded (*p* < 0.05 and < 0.001 respectively) and not blinded (*p* < 0.05) compared to histology

There was a statistically significant difference between the root scores for the PAN and PA images compared to the gold standard, while CBCT values did not differ. Also, blinded and not blinded recordings did not differ statistically. Small resorptions were more difficult to record than large resorptions, except for middle resorptions, i.e., grade 6. Resorption grade 1 was recorded accurately only with CBCT. On the contrary, resorption grade 5 was accurately recorded even on PAN radiographs. Resorption grade 6 was severely underestimated by all techniques. Most of grade 6 resorptions were detected on CBCT. Grade 6 resorptions in the apical third were misdiagnosed on the PA and PAN radiographic images as grade 1 or grade 2 (Fig. 5).

### Comparison of root length as measured by the radiographic techniques

**Fig. 6:**
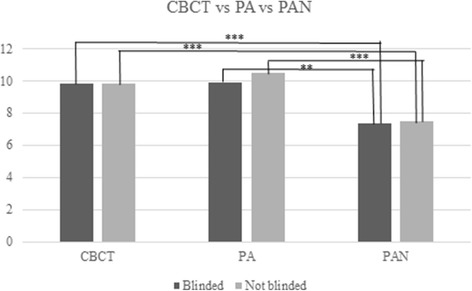
Comparison of linear measurements for CBCT, periapical radiographs (PA), and panoramic radiographs (PAN). PAN differed significantly in both blinded and not blinded (*p* < 0.001) compared to CBCT. Similarly, PAN differed significantly in both blinded (*p* < 0.01) and not blinded (*p* < 0.001) compared to PA

In assessments of linear measures, there was no difference between CBCT and PA in either blinded or not blinded measurements. PAN, however, differed statistically significant from both CBCT and PA, both blinded and not blinded, in this sense (Fig. 6).

### Comparison of modified Malmgren as measured by the radiographic techniques

**Fig. 7:**
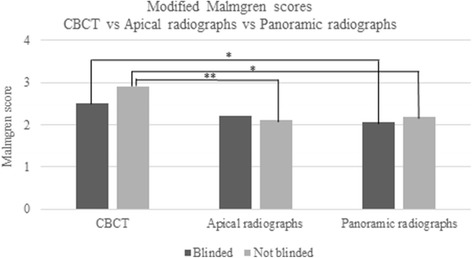
Modified Malmgren scores of CBCT versus periapical radiographs (PA) versus panoramic radiographs (PAN). PAN differed significantly (*p* < 0.05) in the assessment of both blinded and not blinded modified Malmgren score compared to CBCT. Similarly, PA differed significantly in not blinded (*p* < 0.01) compared to CBCT

A statistical significant difference was detected for not blinded PA images compared to CBCT and for both blinded and not blinded PAN compared to CBCT (Fig. 7).

### Inter-examiner reliability

The inter-examiner reliability, as assessed by Cohen’s kappa, differed for the radiographic techniques. For PAN, the kappa value was 1.0, i.e., a perfect agreement. For PA images, the kappa value was 0.71, i.e., a substantial agreement, and, finally, for CBCT, the kappa value was 0.44 demonstrating a moderate agreement. The inter-examiner agreement for the modified Malmgren root index was 0.88, demonstrating a good agreement between the two examiners.

## Discussion

This study shows that CBCT is the most accurate radiographical technique when measuring root length and root resorptions, using a modified Malmgren score, in deciduous maxillary canines. In this study, for the first time, the most common routine radiographs, i.e., PAN and PA radiographs and CBCT, were compared to gold standard histology of true resorptions on extracted teeth.

Orthodontic diagnosis and treatment planning is essentially influenced by radiographic interpretations of the site and severity of root resorptions. Periapical radiographs and PAN are the most commonly used conventional radiographic techniques in orthodontics although they have limitations [1–5]. CBCT, which was presented in the early 1990s, has been showed to be superior to other radiographic methods. However, the diagnostic measures of CBCT requires validation through comparison with different conventional radiographic methods, in addition to a gold standard [8].

To determine the degree of root resorption, different approaches have been employed, being linear measurements, scoring of horizontal root resorption degree, or using scales to describe mesial, distal, buccal, and lingual resorptions [18, 20]. In orthodontic patients, slanted root resorption has been found in up to 15% on the palatal root surfaces as measured with CBCT [21]. Since none of the abovementioned indices describes slanted resorptions, a modified six-scaled Malmgren index for various types of slanted resorption was developed.

A statistically significant difference was found in modified Malmgren scores for PA images and PAN compared to gold standard. On the other hand, CBCT did not differ from gold standard. It was evident that grade 6 resorptions with intact apex were the most difficult to detect, especially on 2D x-rays due to superimposition of structures. Large resorptions were easily interpreted with all techniques, while low resorption grades, especially grade 1 could be interpreted only on CBCT images. Other studies have, as well, showed that CBCT is a superior diagnostic tool in determining location and dimension of small root resorptions compared to PA radiographs [20, 22, 23]. CBCT is also more sensitive than PA images in detecting small simulated lesions drilled on extracted teeth. Small root resorptions less than 0.3 mm can be detected using CBCT while resorptions of 0.6 mm in diameter and 0.3 mm in depth are not detectable on PA radiographs [8, 22, 24].

Furthermore, when linear measurements were assessed and compared to histology, CBCT was once again found to be the most accurate technique. Interestingly, linear root length measurements of the most resorbed side made on PA images were close to gold standard, while the least resorbed side was somewhat overestimated. PAN, however, underestimated the root length in both blinded and non-blinded measurements. Several other studies have also reported the limits with PAN for assessing root resorptions [8, 25, 26].

To overcome the difficulties in identifying tooth structures due to superimposition of 3D structures onto a 2D plane, distortion projection errors, and blurred images, blinded and not blinded assessments of root length and resorptions were performed. This has important clinical relevance since the root resorption detection on the radiograph is considered blinded. Interestingly, the linear root length measurements and index scoring did not differ regardless if it was blinded or not. Even when the assessments were done not blinded, PAN underestimated compared to histology but also when it was compared to CBCT. This clearly demonstrates that PAN is not an accurate method to detect root resorption in the canine region. Similarly, PA differed in assessing resorptions with Malmgren scores; however, in linear measurements, it did not differ. When the different radiographic techniques were compared to each other, PAN was found least accurate in blinded as well as not blinded measurements compared to CBCT and PA [8, 27]. However, none of these studies have compared the results to a gold standard as done in the present study.

The inter-examiner reliability test in the present study showed better agreement with the least accurate radiographic techniques, i.e., PA images and PAN, as it showed lower kappa values with the CBCT which was the most accurate technique. In the literature, other results, with better inter-examiner reliability for CBCT than for both PA and PAN, have been presented. A reason for this difference might be the use of a modified Malmgren score in the present study. This shows that it is easier to have a high agreement between two observers using a less accurate technique [23, 28, 29]. Thus, CBCT can be useful in the detection of small root resorption and to evaluate the severity of the resorption. In addition, CBCT was the technique most accurate in comparison to gold standard, i.e., histology. Moreover, in clinical practice, CBCT gives a 3D image which provides the clinicians with a multitude of data. However, since the radiation exposure of CBCT is higher than the conventional radiographic methods, it is important to consider factors including the probability of obtaining the diagnostic information that is sought from it, its risks, and costs. Therefore, practitioners should always follow the ALARA principle (As Low As Reasonably Achievable), which dictates that every precaution should be taken to minimize radiation exposure to patients [30].

## Conclusions

CBCT is the most accurate technique, both blinded and not blinded, when measuring root length and detecting root resorptions using a modified Malmgren score. PA images were comparable with CBCT for blinded and not blinded root length measurements but were not accurate when assessing root resorptions using the modified Malmgren score. PAN is not a good diagnostic tool for measuring blinded and not blinded root length and root resorptions. A better kappa agreement was seen for the least accurate technique, i.e., PAN.

## References

[1] Elefteriadis JN, Athanasiou AE (1996). Evaluation of impacted canines by means of computerized tomography. Int J Adult Orthodon Orthognath Surg.

[2] Ericson S, Kurol J (1987). Incisor resorption caused by maxillary cuspids: a radiographic study. Angle Orthod..

[3] Ericson S, Kurol J (1988). CT diagnosis of ectopically erupting maxillary canines—a case report. Eur J Orthod.

[4] Peene P, Lamoral Y, Plas H, Wilms G, De Bethune V, Opdebeeck H, Termote JL (1990). Resorption of the lateral maxillary incisor: assessment by CT. J Comput Assist Tomogr.

[5] Stewart JA, Heo G, Glover KE, Williamson PC, Lam EW, Major PW (2001). Factors that relate to treatment duration for patients with palatally impacted maxillary canines. Am J Orthod Dentofac Orthop.

[6] Ericson S, Kurol J (1988). Resorption of maxillary lateral incisors caused by ectopic eruption of the canines: a clinical and radiographic analysis of predisposing factors. Am J Orthod Dentofac Orthop.

[7] Heimisdottir K, Bosshardt D, Ruf S (2005). Can the severity of root resorption be accurately judged by means of radiographs? A case report with histology. Am J Orthod Dentofac Orthop.

[8] Alqerban A, Jacobs R, Souza PC, Willems G (2009). In-vitro comparison of 2 cone-beam computed tomography systems and panoramic imaging for detecting simulated canine impaction-induced external root resorption in maxillary lateral incisors. Am J Orthod Dentofac Orthop.

[9] Almuhtaseb E, Mao J, Mahony D, Bader R, Zhang ZX (2014). Three-dimensional localization of impacted canines and root resorption assessment using cone beam computed tomography. J Huazhong Univ Sci Technolog Med Sci.

[10] Sherrard JF, Rossouw PE, Benson BW, Carrillo R, Buschang PH (2010). Accuracy and reliability of tooth and root lengths measured on cone-beam computed tomographs. Am J Orthod Dentofac Orthop.

[11] Tsai P, Torabinejad M, Rice D, Azevedo B (2012). Accuracy of cone-beam computed tomography and periapical radiography in detecting small periapical lesions. J Endod.

[12] Kullendorff B, Nilsson M, Rohlin M (1996). Diagnostic accuracy of direct digital dental radiography for the detection of periapical bone lesions: overall comparison between conventional and direct digital radiography. Oral Surg Oral Med Oral Pathol Oral Radiol Endod..

[13] Patel S, Dawood A, Wilson R, Horner K, Mannocci F (2009). The detection and management of root resorption lesions using intraoral radiography and cone beam computed tomography—an in vivo investigation. Int Endod J.

[14] Kobayashi K, Shimoda S, Nakagawa Y, Yamamoto A (2004). Accuracy in measurement of distance using limited cone-beam computerized tomography. Int J Oral Maxillofac Implants.

[15] Lund H, Gröndahl K, Gröndahl HG (2009). Accuracy and precision of linear measurements in cone beam computed tomography Accuitomo tomograms obtained with different reconstruction techniques. Dentomaxillofac Radiol..

[16] Pinsky HM, Dyda S, Pinsky RW, Misch KA, Sarment DP (2006). Accuracy of three-dimensional measurements using cone-beam CT. Dentomaxillofac Radiol..

[17] Sakabe J, Kuroki Y, Fujimaki S, Nakajima I, Honda K (2007). Reproducibility and accuracy of measuring unerupted teeth using limited cone beam X-ray CT. Dentomaxillofac Radiol.

[18] Levander E, Malmgren O (1988). Evaluation of the risk of root resorption during orthodontic treatment: a study of upper incisors. Eur J Orthod.

[19] Naoumova J, Kurol J, Kjellberg H (2015). Extraction of the deciduous canine as an interceptive treatment in children with palatal displaced canines-part I: shall we extract the deciduous canine or not?. Eur J Orthod.

[20] Ericson S, Kurol J (2000). Incisor root resorptions due to ectopic maxillary canines imaged by computerized tomography: a comparative study in extracted teeth. Angle Orthod..

[21] Lund H (2011). Cone beam computed tomography in evaluations of some side effects of orthodontic treatment. Swed Dent J Suppl.

[22] Bernardes RA, de Paulo RS, Pereira LO, Duarte MA, Ordinola-Zapata R, de Azevedo JR (2012). Comparative study of cone beam computed tomography and intraoral periapical radiographs in diagnosis of lingual-simulated external root resorptions. Dent Traumatol.

[23] Ren H, Chen J, Deng F, Zheng L, Liu X, Dong Y (2012). Comparison of cone-beam computed tomography and periapical radiography for detecting simulated apical root resorption. Angle Orthod..

[24] Durack C, Patel S, Davies J, Wilson R, Mannocci F (2011). Diagnostic accuracy of small volume cone beam computed tomography and intraoral periapical radiography for the detection of simulated external inflammatory root resorption. Int Endod J.

[25] Gher ME, Richardson AC (1995). The accuracy of dental radiographic techniques used for evaluation of implant fixture placement. Int J Periodontics Restorative Dent.

[26] Sameshima GT, Asgarifar KO (2001). Assessment of root resorption and root shape: periapical vs panoramic films. Angle Orthod.

[27] Alqerban A, Jacobs R, Fieuws S, Willems G (2011). Comparison of two cone beam computed tomographic systems versus panoramic imaging for localization of impacted maxillary canines and detection of root resorption. Eur J Orthod.

[28] Ozen T, Kamburoğlu K, Cebeci AR, Yüksel SP, Paksoy CS (2009). Interpretation of chemically created periapical lesions using 2 different dental cone-beam computerized tomography units, an intraoral digital sensor, and conventional film. Oral Surg Oral Med Oral Pathol Oral Radiol Endod.

[29] Dudic A, Giannopoulou C, Leuzinger M, Kiliaridis S (2009). Detection of apical root resorption after orthodontic treatment by using panoramic radiography and cone-beam computed tomography of super-high resolution. Am J Orthod Dentofac Orthop.

[30] White CS, Pharoah JM. Oral radiology, principles and interpretation. 7th ed. St. Louis: Mosby; 2014.

